# Metabolic effects of basic fibroblast growth factor in streptozotocin-induced diabetic rats: A ^1^H NMR-based metabolomics investigation

**DOI:** 10.1038/srep36474

**Published:** 2016-11-03

**Authors:** Xiaodong Lin, Liangcai Zhao, Shengli Tang, Qi Zhou, Qiuting Lin, Xiaokun Li, Hong Zheng, Hongchang Gao

**Affiliations:** 1 School of Pharmaceutical Sciences, Wenzhou Medical University, Wenzhou 325035, China; 2 Metabonomics Section of Collaborative Innovation Center of Biomedicine, Wenzhou Medical University-Wenzhou University, Wenzhou 325035, China

## Abstract

The fibroblast growth factors (FGFs) family shows a great potential in the treatment of diabetes, but little attention is paid to basic FGF (bFGF). In this study, to explore the metabolic effects of bFGF on diabetes, metabolic changes in serum and feces were analyzed in the normal rats, the streptozocin (STZ)-induced diabetic rats and the bFGF-treated diabetic rats using a ^1^H nuclear magnetic resonance (NMR)-based metabolomic approach. Interestingly, bFGF treatment significantly decreased glucose, lipid and low density lipoprotein/very low density lipoprotein (LDL/VLDL) levels in serum of diabetic rats. Moreover, bFGF treatment corrected diabetes-induced reductions in citrate, lactate, choline, glycine, creatine, histidine, phenylalanine, tyrosine and glutamine in serum. Fecal propionate was significantly increased after bFGF treatment. Correlation analysis shows that glucose, lipid and LDL/VLDL were significantly negatively correlated with energy metabolites (citrate, creatine and lactate) and amino acids (alanine, glycine, histidine, phenylalanine, tyrosine and glutamine). In addition, a weak but significant correlation was observed between fecal propionate and serum lipid (R = −0.35, P = 0.046). Based on metabolic correlation and pathway analysis, therefore, we suggest that the glucose and lipid lowering effects of bFGF in the STZ-induced diabetic rats may be achieved by activating microbial metabolism, increasing energy metabolism and correcting amino acid metabolism.

Diabetes mellitus (DM) is a common metabolic disease characterized by hyperglycemia due to insulin resistance or impaired insulin secretion. DM can cause a series of complications that affect the life quality of more and more people in the world^1^. Guariguata *et al.*^2^ predicted that the number of diabetes is expected to rise to 592 million by 2035. Therefore, it is of great importance to effectively control and manage the prevalence of DM and its complications. Up to now, many strategies have been used for DM treatment, particularly insulin and metformin. However, these two commonly used treatments have side effects; for example, excessive insulin treatment may result in hypoglycemia and metformin can cause body weight loss. Thus, there are increasing demands for new drug discovery to prevent and treat DM.

Fibroblast growth factors (FGFs) represent a large family of polypeptide growth factors that have shown a great potential for DM treatment. Kharitonenkov *et al.*^3^ found that the levels of plasma glucose and triglycerides were decreased to normal levels in both *ob/ob* and *db/db* mice after FGF-21 administration and this effect can be persisted for at least 24 hours. Moreover, FGF-21 did not induce mitogenicity, hypoglycemia and weight change^3^. Wente *et al.*^4^ also reported that FGF-21 can preserve β-cell function and survival and thereby maintain glucose homeostasis. Wu *et al.*^5^ found that amelioration of type 2 diabetes (T2D) can be achieved by antibody-mediated activation of FGFR1, a major functional receptor of FGF-21. In addition, FGF21-mimetic antibody can alleviate diabetes by activating the βKlotho/FGFR1c receptor complex^6^. FGF-19, as another FGFs member, has also shown to improve insulin, glucose and lipid homeostasis in diabetic rodents^7^,^8^. Central injection of FGF-19 in diabetic mice exhibited a glucose-lowering effect^9^,^10^. Recently, a very interesting finding about the therapeutic effect of FGF-1 on T2D was reported by Scarlett *et al.*^11^, where they found that a single central injection of FGF-1 at a dose of 1/10 peripheral injection induces sustained diabetes remission in both mouse and rat models. This antidiabetic effect is achieved by increasing blood glucose clearance into skeletal muscle and liver, and is not accompanied with hypoglycemia and weight loss^11^. Relative to FGF-1, FGF-19 and FGF-21, little attention was paid to the effect of bFGF, also known as FGF-2, on diabetic blood environment. However, bFGF also possesses a potential antidiabetic effect, for example, Rivas-Carrillo *et al.*^12^ found that the transplantation of islets without FGF-2 supplementation failed to control blood glucose level in diabetic mice. Therefore, in the present study, we were interested in how bFGF treatment affected blood metabolism in diabetic rats.

Metabolomics as a relatively new omics technique attempts to analyze a comprehensive set of low-molecular weight metabolites in biomaterials under a particular condition, such as diseases or drug treatment^13^. Nuclear magnetic resonance (NMR) spectroscopy is an attractive technique in metabolomics studies because of its advantages, including simple sample preparation, rapid analysis and high reproducibility. NMR-based metabolomic technique has been used in pharmaceutical research and development^13^,^14^, involving the investigation of pharmacological mechanisms, the evaluation of drug safety and efficacy, and the identification of new targets or biomarkers for drug treatment. In the present study, we analyzed metabolic profiles of serum and feces in the STZ-induced diabetic rats after bFGF treatment and attempted to explore bFGF-induced metabolic effects by using a ^1^H NMR-based metabolomic approach.

## Methods

### Animals

Sprague-Dawley (SD) rats (male, 6 weeks old, body weight = 180–200 g) were obtained from the SLAC Laboratory Animal Co., Ltd. (Shanghai, China) and housed in a specific pathogen-free colony under a fully controlled condition including temperature (23±2 °C), humidity (55±5%) and light (12 h-light-dark cycle and lights on at 8:00 a.m) at the Laboratory Animal Center of Wenzhou Medical University (Wenzhou, China). All rats were given *ad libitum* access to standard rat chow and tap water. In this study, animal care and experimental procedures were strictly in accordance with the Guide for the Care and Use of Laboratory Animals, and approved by the Institutional Animal Care and Use Committee of Wenzhou Medical University. Experiments were reported according to the ARRIVE guidelines.

### STZ-induced diabetic rat model and bFGF treatment

All rats were weighted and randomly divided into the normal control (CON) and diabetic (DM) groups after a 1-week acclimation period. After a 12-h fast, rats in the DM group were received an intraperitoneal (i.p.) injection of streptozotocin (STZ, Sigma-Aldrich) solution at a single dosage of 65 mg/kg of body weight. The STZ solution was prepared in citrate buffer (0.1 M, pH 4.5). Thus, the CON rats were injected with the same volume of sodium citrate. After 3 days, blood glucose level was measured from a tail nick by a handheld glucometer (B/BRAUN omnitest plus). The DM rats were defined and selected when blood glucose level above 16.70 mmol/L. Then, the DM rats were randomly divided into the DM and bFGF groups. Recombinant human bFGF (purity >95%) was purchased from the Grost Biotechnology Co., Ltd. (Wenzhou, China) and prepared in normal saline at a concentration of 3.0 μg/mL. The biological activity of bFGF was validated by the company. In addition, the dose of treatment was suggested by our colleagues according to the pilot study of Zhao *et al.*^15^. Rats in the bFGF group were treated by tail-vein injection of bFGF solution at a dosage of 5 μg/kg of body weight for a 7-day continuous treatment (once daily injection at 8:00 am). Additionally, rats in the CON and DM groups were received the same volume of normal saline. To ensure a complete injection, we started the tail-vein injection after venous blood return was noted at the needle hub.

### Sample collection and extraction

Fecal sample was collected 24 h before rats were sacrificed, frozen in liquid nitrogen immediately, and stored at −80 °C until analysis. After 7 days of bFGF treatment, all rats were fasted overnight and sacrificed by rapid decapitation to avoid stress responses. Blood sample was collected, centrifuged at 3,000 g at 4 °C for 15 min to separate serum, and stored at −80 °C until use. Prior to NMR analysis, frozen feces was pulverized by a stainless steel pulverizer (Redsun electromechanical company LTD. Yongkang, China) and weighed into an Eppendorf tube. Then, phosphate buffer (PBS, pH = 7.4) containing 300 mM sodium chloride was added and extracted by the ultrasonic extraction process for 15 min. The mixture was centrifuged at 5,000 g at 4 °C for 15 min. Then, 400 μL of supernatant was transferred into a 5 mm NMR tube and mixed with 100 μL of D_2_O containing sodium trimethylsilyl propionate-d_4_ (TSP, 0.42 mM) for NMR analysis. In addition, 200 μL of serum sample was thawed and diluted with 250 μL of phosphate buffer (0.2 mM Na_2_HPO_4_/NaH_2_PO_4_, pH = 7.4) and 50 μL of D_2_O. The diluted serum was mixed by vortex and centrifuged at 12,000 g at 4 °C for 15 min. Then, 500 μL of supernatant was transferred into a 5 mm NMR tube for NMR analysis.

### NMR measurement and preprocessing

The ^1^H NMR spectra were measured using a Bruker AVANCE III 600 spectrometer equipped with a triple resonance probe (Bruker BioSpin, Rheinstetten, Germany) at 298 K. A standard single-pulse sequence (ZGPR) with water signal pre-saturation was used to acquire NMR spectra of fecal extract. For serum sample, the Carr-Purcell-Meiboom-Gill (CPMG) pulse sequence with a fixed receiver-gain value was applied to reduce broad NMR peaks from protein and lipid signals. Moreover, the main acquisition parameters were set as follows: spectral width = 12,000 Hz; data points = 256 K; relaxation delay = 4 s; acquisition time = 2.66 s per scan.

The ^1^H NMR spectra were manually corrected for phase and baseline in the Topspin 3.0 software (Bruker BioSpin, Rheinstetten, Germany). The NMR spectra of serum were referenced to the methyl peak of lactate at 1.33 ppm, while the spectra of feces were referenced to TSP peak at 0 ppm, respectively. The ‘icoshift’ procedure was performed to align NMR spectra in MATLAB (R2012a, The Mathworks Inc., Natick, MA, USA)^16^. The spectral region from 0.5 to 8.0 ppm excluding the residual water signals (4.4 to 5.2 ppm) for serum and fecal extract were subdivided and integrated to binning data with a size of 0.01 ppm for further multivariate analysis.

### Multivariate analysis

To discriminate metabolic patterns between different groups, partial least squares-discriminant analysis (PLS-DA) was carried out using Pareto-scaled NMR data in SIMCA 12.0 software (Umetrics, Umeå, Sweden). A leave-one-out (LOO) cross validation method was used for PLS-DA development. Moreover, a permutation test (200 cycles) was conducted to evaluate the performance of PLS-DA, where R^2^ and Q^2^ were calculated as the goodness of fit and the predictive capability of the model, respectively. Generally, these two parameters close to 1.0 represent an excellent model. The PLS-DA score plot exhibits the differences in metabolic patterns between different groups, while the significance of variables was evaluated using the variable importance in the projection (VIP) method. NMR signals were considered important when VIP scores above 1.0 and selected for further analysis.

### Metabolite identification

NMR signals were assigned as shown in Table S1 according to reported data for serum^17^ and feces^18^ as well as the HMDB database^19^. Furthermore, two-dimensional ^13^C-^1^H heteronuclear single quantum coherence (HSQC) experiments were performed on representative samples to verify uncertain assignments. The relative concentrations of identified metabolites were quantified via their peak areas by reference to the internal standard TSP concentration using Chenomx NMR suite 7.7.2 software (Chenomx Inc., Alberta, Canada).

### Statistical analysis

In this study, all rats were randomly assigned to experimental procedures including housing and feeding, animal grouping, STZ injection as well as bFGF treatment. Data acquisition was performed by masking the group of the animals. Differences in metabolite levels between different groups were analyzed with independent-samples T-tests using SPSS software (version 13.0, SPSS), and a statistically significant difference was considered when P value <0.05. Pearson’s correlation between different metabolites and the corresponding P value were calculated using the MATLAB function (‘corrmatrix’, R2012a). Furthermore, correlation heatmap was visualized by the ‘heat map’ module of R software (version 3.3.1, http://www.R-project.org). In the heatmap, cluster analysis was performed with Ward’s method using Euclidean distance and a darker color represents a higher correlation.

## Results

### NMR-based metabolic profiles in serum and feces of rats

**Figure 1:**
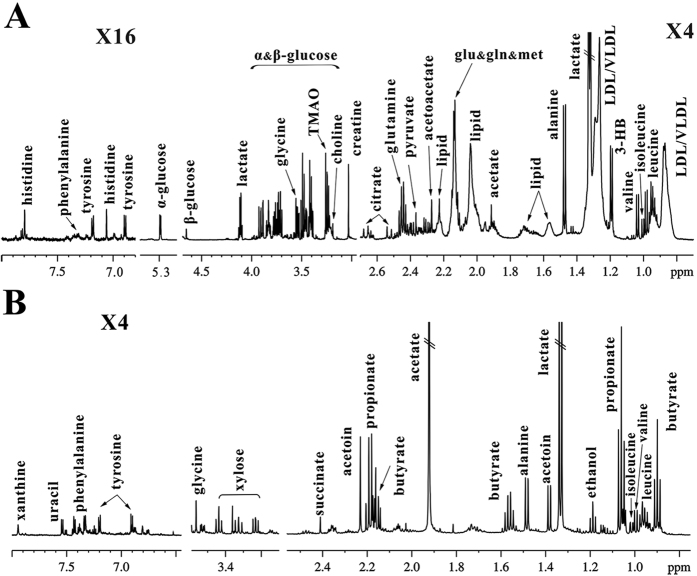
Typical 600 MHz ^1^H NMR spectra of serum (**A**) and feces (**B**) in the normal rats. The detailed assignments of metabolites were shown in Table S1.

Typical ^1^H NMR spectra in serum and feces of the normal rats (control group) are illustrated in Fig. 1A,B, respectively. We identified a series of serum metabolites including energy metabolisms (acetate, citrate, creatine, glucose, lactate and pyruvate), lipid metabolism (lipid and LDL/VLDL), amino acid metabolism (alanine, glutamine, glycine, histidine, isoleucine, leucine, phenylalanine, tyrosine and valine), methylamine metabolism (choline and TMAO) and ketone body metabolism (acetoacetate and 3-hydroxybutyrate). In addition, fecal metabolites mainly include microbial-related metabolites (acetate, butyrate, propionate and acetoin), amino acids (alanine, isoleucine, glycine, leucine, phenylalanine, tyrosine and valine), succinate, ethanol, xylose, uracil and xanthine. The detailed NMR assignment of these metabolites is listed in Table S1.

### Metabolic patterns in serum and feces in diabetic rats treated with bFGF

**Figure 2:**
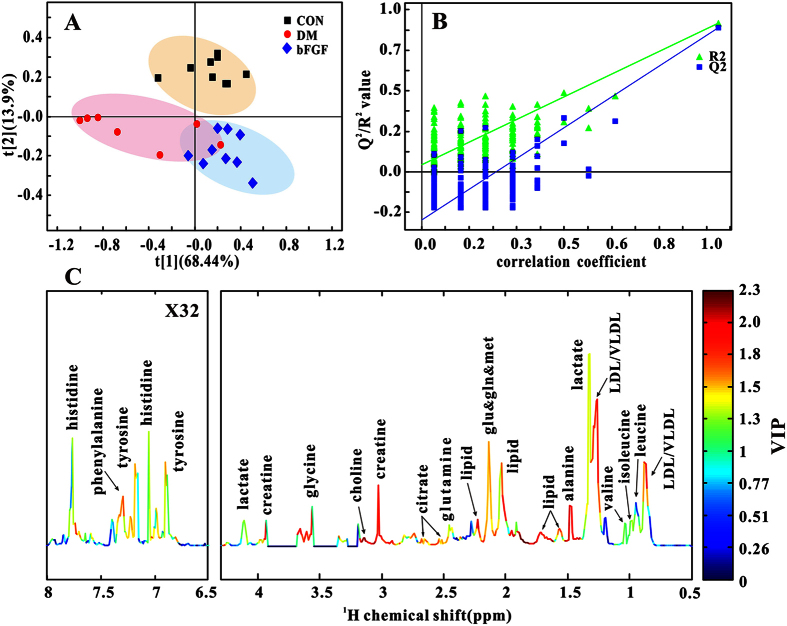
Multivariate analysis based on serum metabolic profiles of the control (CON), diabetic (DM) and bFGF-treated (bFGF) rats: (**A**) PLS-DA score plot. (**B**) Permutation test (200 cycles, R^2^ = 0.880, Q^2^ = 0.854). (**C**) NMR spectrum colored with VIP values.

**Figure 3:**
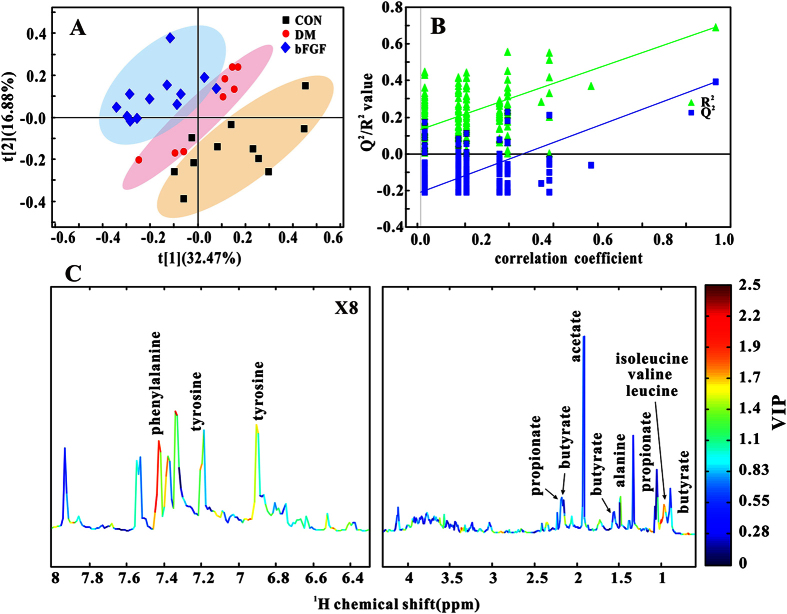
Multivariate analysis based on fecal metabolic profiles of the control (CON), diabetic (DM) and bFGF-treated (bFGF) rats: (**A**) PLS-DA score plot. (**B**) Permutation test (200 cycles, R^2^ = 0.692, Q^2^ = 0.392). (**C**) NMR spectrum colored with VIP values.

In this study, PLS-DA was used to examine changes in metabolic patterns among the CON, DM and bFGF groups as well as identify key metabolites that contributed to metabolic pattern changes. PLS-DA can clearly distinguish among these three groups based on the serum metabolome (Fig. 2A), which is validated by a permutation test (Fig. 2B, R^2^ = 0.880, Q^2^ = 0.854). According to its corresponding VIP plot, a series of metabolites were identified, such as LDL/VLDL, lactate, alanine, creatine, and so on (Fig. 2C). In addition, a clear separation shown in Fig. 3A indicates that faecal metabolic patterns were also different among these three groups (R^2^ = 0.692, Q^2^ = 0.392). Metabolites that contributed to this separation were identified from the VIP plot, involving microbial metabolism (acetate, butyrate and propionate) and amino acid metabolism (alanine, isoleucine, leucine, valine, tyrosine and phenylalanine), as shown in Fig. 3C.

### Metabolic changes in serum and feces in diabetic rats treated with bFGF

Furthermore, these metabolites were relatively quantified and analyzed by correlation and pathway analyses. Metabolic correlation heatmap was illustrated in Figure S1, and the detailed correlations of serum and fecal metabolites with serum glucose, lipid and LDL/VLDL levels were listed in Table 1. We found that glucose, lipid and LDL/VLDL in serum were significantly negatively correlated with alanine, choline, citrate, creatine, glutamine, glycine, histidine, lactate, phenylalanine and tyrosine (Table 1). It can be seen from Table 1 that fecal metabolites were poorly related to serum glucose, lipid and LDL/VLDL, but the correlations of propionate with lipid (R = −0.35, P = 0.046) and LDL/VLDL (R = −0.30, P = 0.054) tended to be statistically significant.

**Figure 4:**
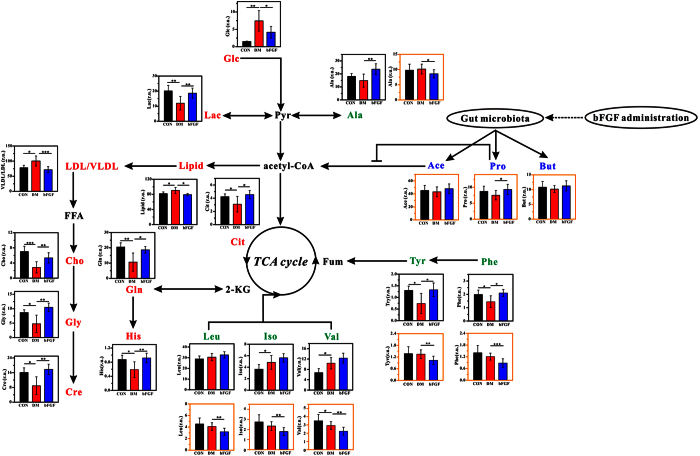
Metabolic changes in rats after bFGF treatment: CON, the control rat (black bar); DM, the diabetic rat (red bar); bFGF, the bFGF-treated rat (blue bar). Red and blue texts indicate metabolites detected from serum and fecal samples, respectively. Green text indicates metabolites detected from both serum (black outline box) and fecal (yellow outline box) samples. Metabolites: 2-KG, 2-ketoglutarate; Glc, glucose; Pyr, pyruvate; Lac, lactate; Ala, alanine; FFA, free fatty acid; Cho, choline; Gly, glycine; Cre, creatine; Cit, citrate; Gln, glutamine; Ace, acetate; Pro, propionate; But, butyrate; Leu, leucine; Iso, isoleucine; Val, valine; Fum, fumarate; Tyr, tyrosine; Phe, phenylalanine; His, histidine. r.u., relative unit. Significant levels: *P < 0.5; **P < 0.01; ***P < 0.001.

Figure 4 shows the metabolic changes among the CON, DM and bFGF groups. We found that the serum levels of glucose, lipid and LDL/VLDL were significantly increased in the DM group compared with the CON group. However, interestingly, there increased levels were significantly reduced after bFGF treatment (Fig. 4). Relative to the normal rats, the DM rats had significantly decreased levels of citrate, choline, creatine, glycine, glutamine, histidine, lactate, phenylalanine and tyrosine in serum, as shown in Fig. 4. It is worth noting that bFGF treatment significantly increased the levels of these metabolites. In addition, fecal SCFAs were also analyzed, although their VIP values were below 1.0. We found that fecal SCFAs levels were increased in the bFGF-treated rats compared with the DM rats, especially propionate (P < 0.05). The DM rats had significantly higher levels of isoleucine and valine in serum than the normal rats, while no significant difference was observed after bFGF treatment. However, faecal BCAAs level was significantly reduced in the bFGF-treated rats compared with the DM rats (Fig. 4). Relative to the DM rats, we also found a significant increase in serum alanine level but a significant decrease in fecal alanine level after bFGF treatment.

## Discussion

The FGF family has shown a great potential in the treatment of diabetes, particularly FGF-1, FGF-19 and FGF-21^20^. However, in this study, we attempted to examine the metabolic effects of bFGF (FGF-2) in the STZ-induced diabetic rats and explore its possible metabolic mechanisms using a ^1^H NMR-based metabolomic approach.

### bFGF reduces serum glucose and lipid level in the STZ-induced diabetic rats

DM is a metabolic disorder characterized by hyperglycemia and hyperlipidemia. In this study, expectedly, the DM rats had significantly increased levels of serum glucose, lipid and LDL/VLDL relative to the normal rats. Interestingly, we found that the serum levels of glucose, lipid, and LDL/VLDL were significantly decreased in the bFGF-treated rats compared with the DM rats. This finding indicates that bFGF may also possess a potential antidiabetic effect, like FGF-1^11^, FGF-19^10^ and FGF-21^6^. Several potential mechanisms linking FGFs and diabetes have been proposed. Hart *et al.*^21^ have reported that attenuation of FGF signaling decreased the number of beta-cells and thereby perturbed glucose homeostasis. The beneficial effect of FGF-21 on glucose homeostasis was implicated in preservation of beta-cell function and survival in diabetic animals^4^. FGF-21 regulated glucose and lipid metabolism via central and peripheral mechanisms^22^. Moreover, adiponectin may also play an important role in the FGF-21-regulated glucose homeostasis in the mouse model^23^. FGF-19 may control glucose metabolism by activating FGF receptor 4^24^, inhibiting gluconeogenesis^25^, increasing insulin-independent glucose disposal^26^ and suppressing the hypothalamic-pituitary-adrenal axis^10^. FGF-19 can also increase fatty acid oxidation^8^. Recently, Scarlett *et al.*^11^ revealed that a single central injection of FGF-1 induced glucose-lowering effect in both mouse and rat models by increasing blood glucose clearance into skeletal muscle and liver. It is worth noting that gut microbiota has been linked with the development of metabolic syndrome including diabetes^27^. However, as far as we known, there is no publication available that reveals the effect of FGFs on gut microbiota. Although we did not directly assess the change of gut microbiota in this study, microbial-related metabolites (short-chain fatty acids, SCFAs) in feces were measured by NMR spectroscopy. We found that fecal SCFAs levels were increased in the bFGF-treated rats compared with the DM rats, especially for propionate (P < 0.05). Fecal SCFAs were negatively correlated with serum lipid and LDL/VLDL levels, and a significant negative correlation was seen between fecal propionate and serum lipid (R = −0.35, P = 0.046). Propionate has been found to inhibit lipid synthesis^28^,^29^. Thus, our results imply that bFGF may regulate lipid metabolism by increasing SCFAs production from gut microbiota. However, the causal relationship still need to be further established.

### bFGF increases energy metabolism in the STZ-induced diabetic rats

DM is always accompanied by abnormal energy metabolism^30^,^31^. In this study, compared with the normal rats, reductions in energy-related metabolites in serum were found in the DM rats, such as citrate, creatine and lactate. After bFGF administration, these metabolites were significantly increased, indicating an increased energy metabolism. FGF-21 has also been shown to enhance energy metabolism^32^,^33^, which plays a vital role in the regulation of glucose and lipid metabolism^34^. Moreover, our results show that the serum levels of glucose, lipid, and LDL/VLDL were significantly negatively correlated with energy-related metabolites including citrate and creatine. Thus, an increase in energy metabolism may be also responsible for the glucose and lipid lowering effects of bFGF treatment. Additionally, Morton *et al.*^26^ revealed that the insulin-independent glucose lowering after a central injection of FGF-19 was implicated in increased glycolysis of glucose to lactate. Interestingly, we also found a significant increase in serum lactate level by tail vein injection of bFGF compared with the DM rats. A significant negative relationship was also observed between glucose and lactate (R = −0.81, P < 0.0001). Thus, we speculate that increased metabolism from glucose to lactate may also contribute to bFGF-induced glucose lowering.

### bFGF corrects amino acid metabolism in the STZ-induced diabetic rats

DM also results in aberrant amino acid metabolism^35^. In this study, the DM rats had significantly lower serum glycine, glutamine, histidine, phenylalanine and tyrosine levels than the normal rats, whereas their levels were significantly increased by bFGF treatment. Glycine is a nonessential amino acid due to its endogenous synthesis in mammals, which can be synthesized from choline^36^. In adult rats, approximately 40–45% of choline is metabolized to form glycine^37^. In this study, therefore, a significant increase in serum glycine was derived from a higher choline level in the bFGF-treated rats relative to the DM rats. Glycine acts a series of physiological functions, for example, protein synthesis for cell growth and creatine production for energy metabolism^36^. Moreover, glycine also plays an important role in lipid digestion and absorption by the conjugation of bile acids^36^. Alvarado-Vasquez *et al.*^38^ reported that dietary glycine supplementation reduced the levels of free fatty acid and triglyceride in the STZ-induced diabetic rats. We also found significant negative correlations of glycine with serum lipid (R = −0.89, P < 0.0001) and LDL/VLDL (R = −0.96, P < 0.0001). Hence, our results indicate that increased glycine level by bFGF treatment may be also associated with the lipid-lowering effect.

Glutamine as another nonessential amino acid has been shown to increase glucose-stimulated insulin secretion from beta-cells^39^. Glutamine is a precursor of glutamate, which plays a key signal link between glucose metabolism and incretin/cAMP action for increasing insulin secretion^40^,^41^. Moreover, Greenfield *et al.*^41^ reported that oral glutamine effectively increases circulating glucagon-like peptide 1, glucose-dependent insulinotropic polypeptide and insulin level in patients with T2D. In this study, glucose was significantly negatively correlated with glutamine (R = −0.92, P < 0.0001) in serum of the DM rats. Therefore, we speculate that the glucose-lowering effect of bFGF may be also attributed to an increased glutamine level.

Histidine is an essential amino acid that can delay diabetic deterioration in the mice model^42^. Furthermore, Feng *et al.*^43^ reported that histidine supplementation improved insulin resistance in obese women with metabolic syndrome by suppressing inflammation. In this study, relative to the normal rats, we found a significantly reduced histidine level in serum of the DM rats. The reduction of serum histidine level was also observed in patients with T2D by Zhang *et al.*^44^. However, serum histidine level was significantly increased in the DM rats after bFGF treatment and negatively correlated with glucose as well as lipid in serum. Taken together, alleviation of DM-induced increases in glucose and lipid by bFGF may be implicated in recovering serum histidine level.

Phenylalanine has been shown to have an insulinotropic effect^45^,^46^. In this study, we found a significant increase in serum phenylalanine level after bFGF treatment as well as a significantly negative correlation between phenylalanine and glucose, indicating that the glucose-lowering effect of bFGF may be associated with an increased serum level of phenylalanine. Tyrosine as a nonessential amino acid is synthesized from phenylalanine. Thus, an identical change in serum tyrosine was observed as compared with phenylalanine. These two amino acids had a close relationship with the development of diabetes^47^. In addition, the reduced levels of amino acids in serum of the DM rats could result in a decrease of energy metabolism, since amino acids can be oxidized to form an alternative energy source and enter TCA cycle. Interestingly, we found that bFGF treatment can recover DM-induced decreases in serum phenylalanine and tyrosine levels. This finding suggests that the potential antidiabetic effect of bFGF may be achieved by correcting amino acid metabolism. However, compared with the DM rats, treatment with bFGF significantly reduced fecal excretion of phenylalanine and tyrosine, while its possible explanation needs to be further investigated.

Increased circulating branched chain amino acids (BCAAs) levels are implicated in insulin resistance and incident T2D^48^,^49^. In the present study, the STZ-induced diabetic rats had a significantly higher BCAAs levels such as isoleucine and valine in serum than the normal rats, indicating that type 1 diabetes (T1D) may also demonstrate increased circulating BCAAs levels. Treatment with bFGF reduced BCAAs levels only in feces, but not in serum, while the possible explanation of this phenomenon still need to be further explored. However, this finding may indicate that the antidiabetic effect of bFGF is not achieved via BCAAs pathway.

In addition, serum alanine level was significantly increased in the bFGF-treated rats compared with the DM rats. Serum alanine accumulation may indicate an inhibition of alanine aminotransferase (ALT), which catalyzes the transfer of an amino group from alanine to α-ketoglutarate and eventually form pyruvate and glutamate. High ALT has been used as a marker of risk for both T1D and T2D^50^,^51^,^52^, so we suggest that the causal relationship between bFGF treatment and ALT needs to be further studied. However, relative to the DM rats, fecal alanine level was significantly decreased after bFGF administration.

## Conclusions

In this study, we found that bFGF treatment can effectively reduce serum glucose and lipid levels in the STZ-induced diabetic rats. Metabolomic results suggest that the glucose and lipid lowering effects may be implicated in activated microbial metabolism, increased energy metabolism as well as corrected amino acid metabolism after bFGF treatment. To our knowledge, this is the first work to investigate the metabolic effects of bFGF in diabetic rats. However, several limitations or further works should be considered: (1) the causal relationship between bFGF treatment and gut microbiota is worth exploring in further work; (2) metabolomic results need to be confirmed using multi-dose bFGF treatments in other animal models; (3) it is of interest to explore whether bFGF level and FGFR signaling can be decreased after STZ induction and recovered by bFGF treatment; (4) a multi-analytical techniques approach is recommended for analyzing more detailed metabolic changes; (5) further work on protein and gene levels will advance understanding of the therapeutic effect of bFGF on diabetes and its potential mechanisms. In addition, care should be taken in clinical application of bFGF as it can induce mitogenicity. Translational studies are encouraged to modify it and facilitate its application for the treatment of diabetes in humans.

## Additional Information

**How to cite this article**: Lin, X. *et al.* Metabolic effects of basic fibroblast growth factor in streptozotocin-induced diabetic rats: A ^1^H NMR-based metabolomics investigation. *Sci. Rep.* **6**, 36474; doi: 10.1038/srep36474 (2016).

**Publisher’s note:** Springer Nature remains neutral with regard to jurisdictional claims in published maps and institutional affiliations.

## Supplementary Material

Supplementary Information

## Figures and Tables

**Table 1:** Correlations of serum and fecal metabolites with serum glucose, lipid and LDL/VLDL levels^a^.

Sample	Metabolite	Glucose		Lipid		LDL/VLDL^b^	
		R	P	R	P	R	P
Serum	alanine	−0.47	0.0045	−0.77	<0.0001	−0.81	<0.0001
	choline	−0.88	<0.0001	−0.68	<0.0001	−0.73	<0.0001
	citrate	−0.76	<0.0001	−0.82	<0.0001	−0.91	<0.0001
	creatine	−0.78	<0.0001	−0.94	<0.0001	−0.95	<0.0001
	glutamine	−0.92	<0.0001	−0.85	<0.0001	−0.91	<0.0001
	glycine	−0.75	<0.0001	−0.89	<0.0001	−0.96	<0.0001
	histidine	−0.76	<0.0001	−0.85	<0.0001	−0.90	<0.0001
	isoleucine	0.12	0.49	−0.35	0.04	−0.37	0.03
	lactate	−0.81	<0.0001	−0.89	<0.0001	−0.82	<0.0001
	leucine	0.07	0.69	−0.29	0.09	−0.33	0.05
	phenylalanine	−0.67	<0.0001	−0.86	<0.0001	−0.90	<0.0001
	tyrosine	−0.85	<0.0001	−0.89	<0.0001	−0.93	<0.0001
	valine	0.30	0.08	−0.22	0.21	−0.24	0.16
Feces	acetate	0.03	0.87	−0.11	0.54	−0.13	0.45
	alanine	0.08	0.63	0.25	0.14	0.26	0.13
	butyrate	−0.08	0.66	−0.17	0.33	−0.18	0.28
	isoleucine	−0.20	0.25	0.12	0.50	0.13	0.44
	leucine	−0.16	0.37	0.16	0.35	0.19	0.28
	phenylalanine	−0.19	0.27	0.11	0.51	0.14	0.41
	propionate	−0.18	0.31	−0.35	0.046	−0.30	0.054
	tyrosine	−0.02	0.90	0.19	0.28	0.22	0.20
	valine	−0.23	0.18	0.15	0.40	0.15	0.39

^a^data were presented as Pearson’s correlation coefficient (R) and its statistical significance (P), N = 35; ^b^low-density/very-low-density lipoprotein.
