# Supplementary material for: Metabolic effects of basic fibroblast growth factor in streptozotocin-induced diabetic rats: A 1H NMR-based metabolomics investigation
Source: Sci Rep. 2016 Nov 3;6:36474. doi: 10.1038/srep36474 (PMC5093531; doi:10.1038/srep36474)
Supplement: Supplementary Information [file srep36474-s1.doc]

**Metabolic effects of basic fibroblast growth factor in** **streptozotocin-induced diabetic rats: A ^1^H NMR-based metabolomics investigation**

Xiaodong Lin^1^, Liangcai Zhao^1,2^, Shengli Tang^1^, Qi Zhou^1^, Qiuting Lin^1^, Xiaokun Li^1,2^, Hong Zheng^1,2,*^, and Hongchang Gao^1,2,*^

^1^School of Pharmaceutical Sciences, Wenzhou Medical University, Wenzhou 325035, China

^2^Metabonomics Section of Collaborative Innovation Center of Biomedicine, Wenzhou Medical University-Wenzhou University, Wenzhou 325035, China

*Corresponding author: Tel.: +86 577 86699715; E-mail: gaohc27@wmu.edu.cn (H.C. Gao); 123zhenghong321@163.com (H. Zheng).

**Abstract**

In the supporting material, major metabolite signaling in ^1^H NMR spectra obtained from serum and feces in rats were assigned using published data and the HMDB database (Table S1). Results based on correlation heatmap between different metabolites in serum and feces of rats are illustrated in Figure S1.

**Table S1.** Assignment of metabolites in ^1^H NMR spectra from serum and feces samples in the normal rats.

| No. | Compound | ppm | Assignment | Multiplicity | Sample |
| --- | --- | --- | --- | --- | --- |
| 1 | LDL/VLDL | 0.8-0.9, 1.22-1.3 | CH_3_(CH_2_)_n_/CH_3_CH_2_CH_2_C=, (CH_2_)_n_/CH_2_CH_2_CH_2_CO | m, m | S |
| 2  3  4 | Leucine  Isoleucine  Valine | 0.95, 0.96  1.01  1.03 | δCH_3_, δ’CH_3_  δCH_3_  γ’CH_3_ | d, d  d  d | S,F  S,F  S,F |
| 5 | 3-HB | 1.19 | γCH_3_ | d | S |
| 6 | Lactate | 1.33, 4.11 | βCH_3_, αCH | d, q | S,F |
| 7 | Alanine | 1.47 | βCH_3_ | d | S,F |
| 8 | Acetate | 1.91 | CH_3_ | s | S,F |
| 9 | Lipid | 1.57, 1.7, 2.03, 2.23 | CH_2_CH_2_CO, CH_2_CH_2_C=C, CH_2_C=C, CH_2_CO | m, m, m, m | S |
| 10 | Glu&Gln&Met | 2.1-2.18 | various | m | S |
| 11 | Acetoacetate | 2.27 | CH_3_ | s | S |
| 12 | Pyruvate | 2.37 | βCH_3_ | s | S |
| 13 | Glutamine | 2.14, 2.45 | αCH, βCH_2_ | m, m | S |
| 14 | Citrate | 2.54, 2.68 | half CH_2_, half CH_2_ | d, d | S |
| 15 | Creatine | 3.03 | CH_2_ | s | S |
| 16 | Choline | 3.18 | N(CH_3_)_3_ | s | S |
| 17 | TMAO | 3.26 | N(CH_3_)_3_ | s | S |
| 18 | α&β-glucose | 3.2-3.55, 3.69-3.91 | various | m, m | S |
| 19 | Glycine | 3.56 | CH_2_ | s | S,F |
| 20 | β-glucose | 4.65 | H1 | d | S |
| 21 | α-glucose | 5.23 | H1 | d | S |
| 22 | Tyrosine | 6.9, 7.18 | CH, CH | dd, dd | S,F |
| 23 | Phenylalanine | 7.3, 7.37, 7.42 | H2 & H6, H4, H3 & H5 | m, m, m | S,F |
| 24 | Histidine | 7.06, 7.8 | CH, CH | s, s | S |
| 25 | Butyrate | 0.9, 1.56, 2.15 | CH_3_, CH_2_, CH_2_ | t, m, t | F |
| 26 | Propionate | 1.06, 2.19 | CH_3_, CH_2_ | t, m | F |
| 27 | Acetoin | 1.38, 2.23 | CH_3_, CH_3_ | d, s | F |
| 28 | Succinate | 2.41 | CH_2_ | s | F |
| 29 | Ethanol | 1.19 | CH_3_ | t | F |
| 30 | Xylose | 3.25, 3.33, 3.42 | various | dd, dd, t | F |
| 31 | Uracil | 7.54 | CH | d | F |
| 32 | Xanthine | 7.94 | CH | s | F |

Abbreviation: S, serum; F, feces; s, singlet; d, doublet; t, triplet; q, quartet; dd, double doublet; m, multiplet; LDL/VLDL, low-density/very-low-density lipoprotein; BCAAs, branched chain amino acids; 3-HB, 3-hydroxybutyrate; Glu, glutamate; Gln, glutamine; Met, methionine; TMAO, *N*-oxide trimethylamine.

**
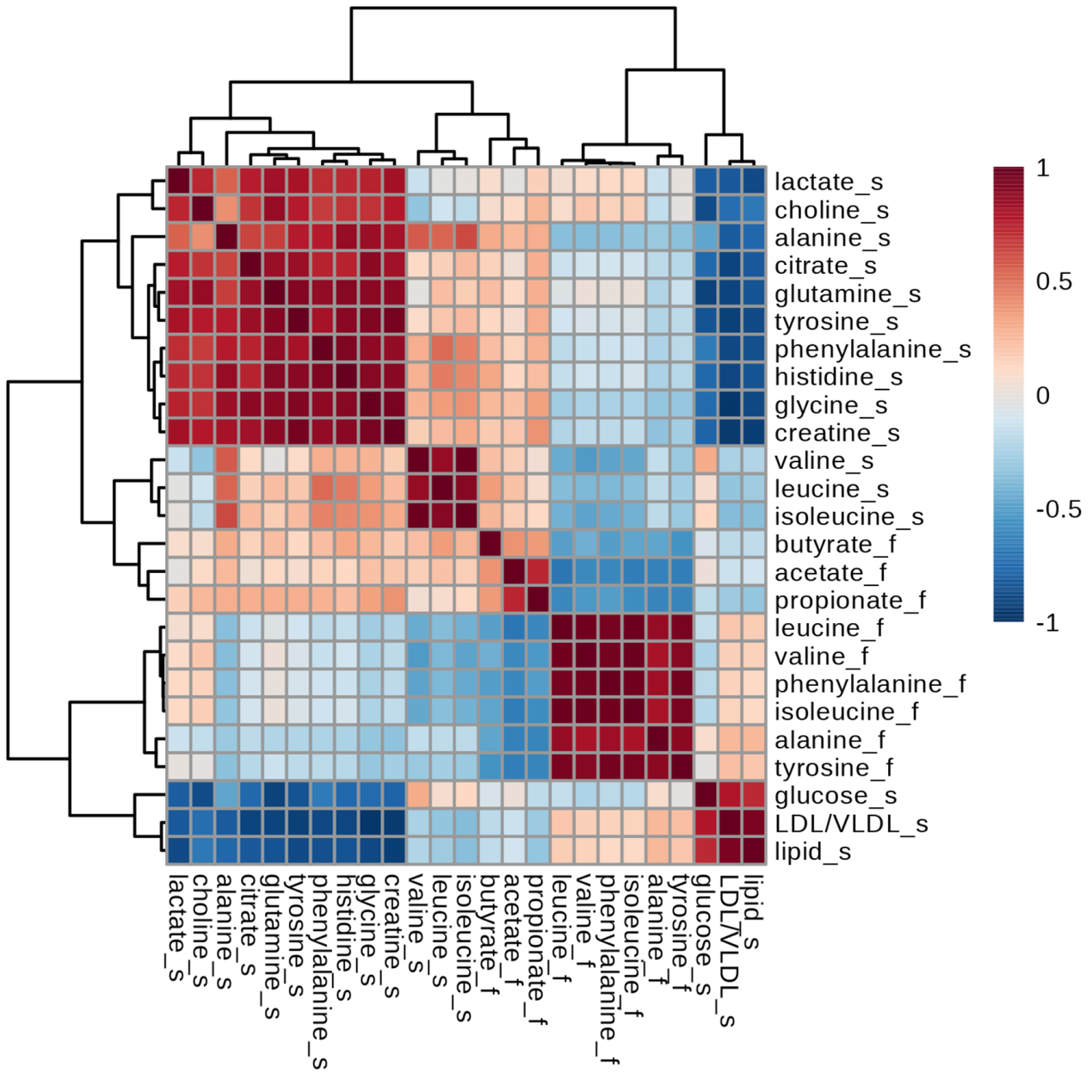
Figure S1.** Correlation heatmap between different metabolites obtained from serum (s) and feces (f) in rats.
